# Comparative Genomics and *in vitro* Infection of Field Clonal Isolates of *Brucella melitensis* Biovar 3 Did Not Identify Signature of Host Adaptation

**DOI:** 10.3389/fmicb.2018.02505

**Published:** 2018-10-22

**Authors:** Marion Holzapfel, Guillaume Girault, Anne Keriel, Claire Ponsart, David O’Callaghan, Virginie Mick

**Affiliations:** ^1^EU/OIE/FAO and National Reference Laboratory for Brucellosis, Animal Health Laboratory, Anses/Paris-Est University, Maisons-Alfort, France; ^2^VBMI, INSERM, U1047, Université de Montpellier, Nîmes, France; ^3^CNR Laboratoire Expert Brucella, Service de Microbiologie, CHU Caremeau, Nîmes, France

**Keywords:** brucellosis, *Brucella melitensis*, host preference, whole genome sequencing, comparative genomics, macrophage infection, adaptation

## Abstract

*Brucella* spp. are responsible for brucellosis, a widespread zoonosis causing reproductive disorders in animals. Species-classification within this monophyletic genus is based on bacteriological and biochemical phenotyping. Traditionally, *Brucella* species are reported to have a preferential, but not exclusive mammalian host. However, this concept can be challenged since many *Brucella* species infect a wide range of animal species. Adaptation to a specific host can be a driver of pathogen variation. It is generally thought that *Brucella* species have highly stable and conserved genomes, however the degree of genomic variation during natural infection has not been documented. Here, we investigated potential genetic diversity and virulence of *Brucella melitensis* biovar 3 field isolates obtained from a single outbreak but from different host species (human, bovine, small ruminants). A unique MLVA-16 pattern suggested all isolates were clonal. Comparative genomic analyses showed an almost non-existent genetic diversity among isolates (only one SNP; no architectural rearrangements) and did not highlight any signature specific to host adaptation. Similarly, the strains showed identical capacities to enter and replicate in an *in vitro* model of macrophage infection. In our study, the absence of genomic variability and similar virulence underline that *B. melitensis* biovar 3 is a broad-host-range pathogen without the need to adapt to different hosts.

## Introduction

Brucellosis, caused by bacteria of the *Brucella* genus, is a widespread zoonosis causing reproductive disorders in animals and a debilitating infection in humans. *Brucella* classification is based on bacteriological and biochemical phenotypes (Ficht, 2010). Traditionally, different species were reported to have a preferential mammalian host, however in reality, spill over into a wide range of animal species has been reported. *B. melitensis*, for example, primarily responsible for disease in domestic small ruminants (sheep and goats), also causes natural infections in bovids, camelids and wild ruminants (e.g., ibex) (Office International des Epizooties [OIE], 2016) (Garin-Bastuji et al., 2014), and is the major species responsible for human infections.

Comparative genomics have allowed us to identify the key steps in the evolution of *Brucella* from a soil organism to a stealth pathogen. This has involved the acquisition of a battery of virulence factors followed by a toning down of virulence (Wattam et al., 2014). What is not understood is how the different species have adapted to their ‘preferential’ host, or the time line of this adaptation. Despite the strong sequence homogeneity within the genus (>94% nucleotide identity) (Wattam et al., 2009), genome-wide studies suggest genetic variation, especially pseudogenization, i.e., due to *IS*711 insertion and point mutations within marine *Brucella* genomes (Suárez-Esquivel et al., 2017), might play a key role in *Brucella* host preference as the bacteria evolved with their respective preferential hosts (Chain et al., 2005). In a recent report, Ke and collaborators suggested that *B. melitensis* rapidly accumulates mutations during *in vivo* passage, however this observation is not supported by the high levels of sequence conservation seen between the genome sequences available in Genbank (Ke et al., 2012).

In this study we investigate seven clonal isolates of *B*. *melitensis* biovar 3 (bv3) isolated from different mammalian hosts during a well characterized outbreak in southern France. We used *in vitro* virulence assays and comparative genomics to determine whether genetic variation is required for the ability to infect different hosts.

## Materials and Methods

### Bacterial Strains

Seven isolates (Supplementary Table S1) collected over a 1-year period (2000–2001) from a restricted geographical area (Drôme, South-East France) from different host species: human (*n* = 1); bovine (*n* = 2); small ruminants (sheep and goat) (*n* = 4) were biotyped using standard procedures (Office International des Epizooties [OIE], 2016). Records regarding this epidemiological event available at the National Reference Laboratory (NRL) for brucellosis were examined. At least one abortion occurred amongst infected animals, probably in small ruminant. Epidemiological and serological investigations conducted to the slaughtering of the herd.

*B. melitensis* reference strains 16M (bv1) and Ether (bv3) (Supplementary Table S1) were included in this study.

### Molecular Analyses and Comparative Genomics

Genomic DNA was extracted using High Pure DNA Template Preparation kit (Roche Diagnostics, France), according to the manufacturer’s recommendations. Real Time-PCR and MLVA-16 assays were performed as previously described (Al Dahouk et al., 2007; Bounaadja et al., 2009).

Genome sequencing was performed on an Illumina HiSeq2500 platform (2 × 250 bp paired-end reads). Raw reads were trimmed using Trimmomatic (Bolger et al., 2014) and quality controls were conducted with FastQC (Andrews, 2010). Mapping assemblies against reference concatenated genomes (i.e., both chromosomes I and II are concatenated together in a single file) of Ether and 16M were performed using Bionumerics v7.6.2 [Burrows-Wheeler Aligner BWA (Li and Durbin, 2009)], as well as *de novo* assembly using SPAdes v3.9. Annotations were performed with Prokka (Seemann, 2014) and alignments with progressiveMAUVE (Darling et al., 2010) and BLAST Ring Image Generator (BRIG) 0.95 (Alikhan et al., 2011). Genome assemblies were evaluated with QUAST 4.6.3 (Gurevich et al., 2013).

Single Nucleotide Polymorphism (SNP) and clustering (maximum-parsimony) analyses were performed using Bionumerics v7.6.2 (wgSNP-module). Applied filters removed indels, repeated regions and rRNAs with minimum ten bp-distance between SNPs. Non-synonymous, synonymous and intergenic SNPs were identified against 16M. Local alignment of interesting regions were done with ClustalW (Thompson et al., 1994) via MEGA 6 (Tamura et al., 2013).

To functionally analyze genomes, Roary v3.6.1 (Page et al., 2015) was used to generate matrices of presence/absence of core (i.e., genes present in all genomes included in the study) and accessory (i.e., not core genes) genes among seven investigated genomes, or among seven genomes vs. 16M. Scoary (Brynildsrud et al., 2016) was used to associate the presence/absence of genes/SNPs to given clustering according to host-range.

Sequences were deposited in European-Nucleotide-Archive (Study PRJEB26921) (Supplementary Table S1).

### Macrophage Infection Assays

Murine J774.1 macrophages (ATCC^®^ TIB-67^TM^) were cultivated and infected with *Brucella* using a standard gentamycin protection protocol as described previously (Soler-Llorens et al., 2016). All experiments were performed in triplicate and independently repeated three times.

The ability of infection of different field isolates was represented as the *Brucella* penetration index (BPI) and the *Brucella* multiplication index (BMI). BPI was defined as the percentage of the inoculum inside macrophages at 2 h post-inoculation (PI); BMI as the ratio of CFU in cell lysates at 48 h PI to CFU at 2 h PI. Results were analyzed using a non-parametric Kruskal-Wallis test (GraphPad Prism program) with significant *p*-values < 0.05.

## Results

Seven investigated isolates were confirmed as *B. melitensis* bv3 using classical microbiological and molecular methods. All seven isolates had identical phenotypes. All isolates had an identical MLVA-16 pattern: Bruce06 3U; Bruce08 5U; Bruce11 3U; Bruce12 13U; Bruce42 1U; Bruce43 1U; Bruce45 3U; Bruce55 3U; Bruce18 7U; Bruce19 42U; Bruce21 8U; Bruce04 8U; Bruce07 5U; Bruce09 12U; Bruce16 8U; Bruce30 3U. This suggests that the isolates were clonal.

### Comparative Genomics

**FIGURE 1:**
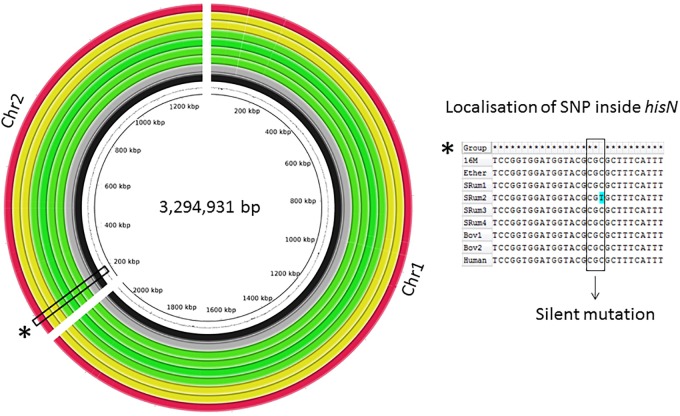
Circular alignment representation of investigated *Brucella melitensis* bv3 genomes. Genomes of seven clonal *B. melitensis* bv3 isolates were *de novo* assembled. BRIG alignment of investigated genomes and Ether against 16M are shown. Rings from inside to outside: GC-% of 16M, 16M (black), Ether (gray), *B. melitensis* bv3 isolated from small ruminants (*n* = 4) (green), from bovine (*n* = 2) (yellow) and from human (*n* = 1) (red). Alignment of the *hisN* region flanking the unique SNP between seven isolates generated with ClustalW.

The global genomic architecture of the seven strains was consistent with those of published *B. melitensis* genomes (i.e., presence of two chromosomes). The field isolates had a genome size of approximately 3.3 Mbp, a G-C content similar to that of the Ether genome (57.24% vs. 57.22%) with 3,210–3,218 predicted protein-encoding genes (QUAST). After filtering, a total of 2,748 SNPs were identified among all studied strains vs. 16M, but only one SNP was observed amongst the seven field strains, in an ovine isolate (Figure 1). This C-to-T substitution at position 267 of the *hisN* gene coding for histidinol-phosphatase (BME_RS10415) is a silent mutation. Manual analysis also showed a bovine isolate harbored another SNP in *hisN* (A-to-G at nucleotide 599) that was discarded by automated analysis; this lead to an Asp200Gly modification of HisN.

Genome-wide comparison from *de novo* assemblies showed a perfect collinearity of the seven genomes as well as with those of 16M and Ether, highlighting absence of genomic rearrangements (Figure 1). Further comparison regarding the pan-genome, i.e., among seven genomes, highlighted a total amount of 3,196 predicted genes, including 3,141 core genes and 55 accessory genes. The strains showed a full complement of previously described virulence factors (He, 2012).

### Macrophage Infection Assays

**FIGURE 2:**
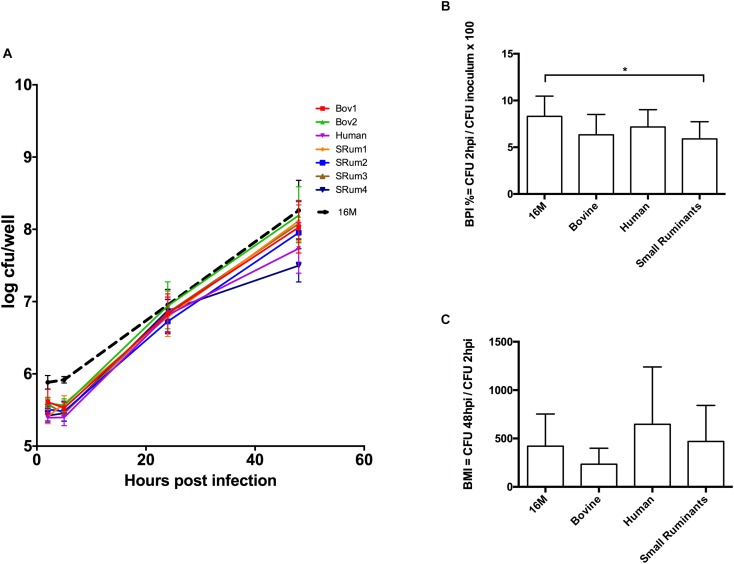
Macrophage infection assay with clonal *B. melitensis* bv3 isolates from different host-range. **(A)** Intracellular growth of *Brucella* isolates inside murine macrophages J774A.1. Each point is performed in triplicates from three independent experiments and represents the log_10_ of the mean ± SD CFU/well. **(B)** Adhesion/Invasion capacity of *Brucella* isolates. **(C)** *Brucella* multiplicity index. Isolates are grouped into three host-categories – bovine (*n* = 2), human (*n* = 1) and small ruminants (*n* = 4)– and compared to the reference *B. melitensis* biovar 1str. 16M. Error bars denote standard errors. Statistical significance of each group was determined by Kruskal-Wallis test. ^∗^*p* < 0.05. Bov1/2, *B. melitensis* bv3 isolated from bovine; Human, *B. melitensis* bv3 isolated from human; SRum1/2/3/4, *B. melitensis* bv3 isolated from small ruminants.

The virulence of the isolates was compared within an *in vitro* murine macrophage infection model. The seven bv3 isolates showed similar ability to infect and multiply in macrophages as the reference strain. In our conditions, *B. melitensis* bv3 small ruminant isolates had slightly but significantly lower BPI values than 16M (*p* = 0.02), unlike human and bovine isolates (*p* > 0.99 and *p* = 0.19, respectively) (Figure 2B). BPI values showed no significant difference among isolates whatever host-species (*p* = 0.34 for human vs. small ruminants to >0.99 for other comparisons) (Figure 2B). At 48 h PI, BMI observed between 16M vs. isolates (*p* > 0.99 for all comparisons) and among isolates did not show significant difference (*p* = 0.26 bovine vs. human; *p* = 0.37 bovine vs. small ruminants; *p* > 0.99 human vs. small ruminants) correlated with host-species (Figure 2C). Indeed, multiplication inside macrophages was similar to the intracellular growth of reference 16M (Figure 2A), and statistically identical among tested isolates.

## Discussion

*Brucella* species have been classically assigned to preferential, but not exclusive animal hosts. Hence, *B. melitensis* is associated with infection of small ruminants and *B. abortus* with cattle. There have, however been numerous reports of *B. melitensis* causing abortion in cattle (Verger et al., 1989; Alvarez et al., 2011; El-Diasty et al., 2018). In this study, we characterized seven *B. melitensis* bv3 field isolates, considered as epidemiologically related because isolated from a same outbreak (same geographical area, same time-period), but from different mammalian hosts. As it was not possible to distinguish between the strains phenotypically and the strains all had the same single identical pattern in MLVA-16, we considered the seven strains as clonal, according to the definition of a “clone” from Tenover et al. (1995) isolates that are indistinguishable from each other by a variety of genetic tests (e.g., PFGE, multilocus enzyme electrophoresis, or ribotyping) or that are so similar that they are presumed to be derived from a common parent. Consequently, this study mimics an *in vivo* experimental infection aiming to determine whether genetic modifications are induced during the short-time adaptation of *B. melitensis* to a transition of environment, i.e., different host species, host being considered as driver of pathogen variation. Adaptation is defined as the process of change by which an organism or species becomes better suited to its environment (Ryall et al., 2012).

Comparison with the genomes of two *B. melitensis* reference strains showed perfect synteny. Our in-depth comparative genomics analyses revealed an almost non-existent genetic diversity among the seven *Brucella* field isolates, with only one SNP confirming that there is no signature of adaptation to different host-species. Most importantly, our data is in complete contrast with the study from Ke et al. (2012) who reported over 5,000 SNPs after 13 weeks mouse passage of *B. melitensis* 16M and suggested that *Brucella* accumulates a large number of adaptive mutations during chronic infection.

The seven field isolates showed identical virulence in murine macrophages. *B. melitensis* clearly has the intrinsic capacity to infect a wide range of mammalian hosts without the need to adapt. It is more likely that the traditional host preference was due to the epidemiology and lack of contact between infected small ruminants and other livestock. Domestic dogs and cats, and feral swine are occasionally infected with *Brucella* through contact with infected livestock (Stoffregen et al., 2007; Lucero et al., 2008; Hinic et al., 2010; Wareth et al., 2017). *B. melitensis* bv3 has also been isolated from Nile catfish, probably infected by contaminated animal waste in the water contamination, showing the broad spectrum of hosts (El-Tras et al., 2010). Some *Brucella* species have a more restricted host range. *B. suis* bv2, which is endemic in wild boar and hares in much of Europe, shows low levels of pathogenicity for ruminants and for humans (Mailles et al., 2017). Genome analysis showed a large inversion and a number of INDELs and SNPs, although none were predicted to affect known virulence factors (Ferreira et al., 2017). The clearest example of host restriction is *B. ovis*, which is almost only pathogenic for sheep, with some studies reporting clinical sign in Red Deer (Ridler et al., 2012). Here a clear process of genome degradation and accumulation of pseudogenes is evident (Tsolis et al., 2009). Genomic decay seems to be associated with a host-restricted pattern, whereas intact genes are present in broad-host-range pathogens (Kirzinger and Stavrinides, 2012). The absence of pseudogenes and/or of genomic rearrangement among genomes studied here underlines that *B. melitensis* bv3 remains a broad-host-range pathogen.

In conclusion, this study shows that *B. melitensis* does not require any adaptive mutations when infecting different mammalian hosts. We cannot exclude the possibility that adaptation to a different host requires differential expression of specific genes that cannot be detected with the simple experimental models that we use.

## Author Contributions

VM and DO conceived the study. CP obtained funding. MH and GG performed genomics analyses. MH and AK performed macrophage infection assays. MH, GG, CP, and VM performed data interpretation. MH, DO, and VM wrote the paper. All authors read and approved the manuscript content.

## Conflict of Interest Statement

The authors declare that the research was conducted in the absence of any commercial or financial relationships that could be construed as a potential conflict of interest.

## Supplementary Material

TABLE S1 Field *Brucella* isolates investigated in this study and reference strains from public databases.

## References

[B1] Al Dahouk S. Fleche P. L. Nockler K. Jacques I. Grayon M. Scholz H. C. (2007). Evaluation of *Brucella* MLVA typing for human brucellosis.*J. Microbiol. Methods* 69 137–145. 10.1016/j.mimet.2006.12.015 17261338

[B2] Alikhan N. F. Petty N. K. Ben Zakour N. L. Beatson S. A. (2011). BLAST ring image generator (BRIG): simple prokaryote genome comparisons. *BMC Genomics* 12:402. 10.1186/1471-2164-12-402 21824423 PMC3163573

[B3] Alvarez J. Saez J. L. Garcia N. Serrat C. Perez-Sancho M. Gonzalez S. (2011). Management of an outbreak of brucellosis due to *B. melitensis* in dairy cattle in Spain. *Res. Vet. Sci.* 90 208–211. 10.1016/j.rvsc.2010.05.028 20579679

[B4] Andrews S. (2010). *FastQC: A Quality Control tool for High Throughput Sequence Data*. Available at: http://www.bioinformatics.babraham.ac.uk/projects/fastqc

[B5] Bolger A. M. Lohse M. Usadel B. (2014). Trimmomatic: a flexible trimmer for illumina sequence data. *Bioinformatics* 30 2114–2120. 10.1093/bioinformatics/btu170 24695404 PMC4103590

[B6] Bounaadja L. Albert D. Chenais B. Henault S. Zygmunt M. S. Poliak S. (2009). Real-time PCR for identification of *Brucella* spp.: a comparative study of IS711, bcsp31 and per target genes. *Vet. Microbiol.* 137 156–164. 10.1016/j.vetmic.2008.12.023 19200666

[B7] Brynildsrud O. Bohlin J. Scheffer L. Eldholm V. (2016). Rapid scoring of genes in microbial pan-genome-wide association studies with Scoary. *Genome Biol.* 17:238. 10.1186/s13059-016-1108-8 27887642 PMC5124306

[B8] Chain P. S. Comerci D. J. Tolmasky M. E. Larimer F. W. Malfatti S. A. Vergez L. M. (2005). Whole-genome analyses of speciation events in pathogenic *Brucellae*. *Infect. Immun.* 73 8353–8361. 10.1128/IAI.73.12.8353-8361.2005 16299333 PMC1307078

[B9] Darling A. E. Mau B. Perna N. T. (2010). Progressivemauve: multiple genome alignment with gene gain, loss and rearrangement. *PLoS One* 5:e11147. 10.1371/journal.pone.0011147 20593022 PMC2892488

[B10] El-Diasty M. Wareth G. Melzer F. Mustafa S. Sprague L. D. Neubauer H. (2018). Isolation of *Brucella abortus* and *Brucella melitensis* from seronegative cows is a serious impediment in brucellosis control. *Vet. Sci.* 5:E28. 10.3390/vetsci5010028 29522464 PMC5876578

[B11] El-Tras W. F. Tayel A. A. Eltholth M. M. Guitian J. (2010). *Brucella* infection in fresh water fish: evidence for natural infection of Nile catfish, *Clarias gariepinus*, with *Brucella melitensis*. *Vet. Microbiol.* 141 321–325. 10.1016/j.vetmic.2009.09.017 19880265

[B12] Ferreira A. C. Correa de Sa M. I. Dias R. Tenreiro R. (2017). MLVA-16 typing of *Brucella suis* biovar 2 strains circulating in Europe. *Vet. Microbiol.* 210 77–82. 10.1016/j.vetmic.2017.09.001 29103700

[B13] Ficht T. A. (2010). *Brucella* taxonomy and evolution. *Future Microbiol.* 5 859–866. 10.2217/fmb.10.52 20521932 PMC2923638

[B14] Garin-Bastuji B. Hars J. Drapeau A. Cherfa M. A. Game Y. Le Horgne J. M. (2014). Reemergence of *Brucella melitensis* in wildlife, France. *Emerg. Infect. Dis.* 20 1570–1571. 10.3201/eid2009.131517 25152274 PMC4178400

[B15] Gurevich A. Saveliev V. Vyahhi N. Tesler G. (2013). QUAST: quality assessment tool for genome assemblies. *Bioinformatics* 29 1072–1075. 10.1093/bioinformatics/btt086 23422339 PMC3624806

[B16] He Y. (2012). Analyses of *Brucella* pathogenesis, host immunity, and vaccine targets using systems biology and bioinformatics. *Front. Cell Infect. Microbiol.* 2:2. 10.3389/fcimb.2012.00002 22919594 PMC3417401

[B17] Hinic V. Brodard I. Petridou E. Filioussis G. Contos V. Frey J. (2010). Brucellosis in a dog caused by *Brucella melitensis* rev 1. *Vet. Microbiol.* 141 391–392. 10.1016/j.vetmic.2009.09.019 19828267

[B18] Ke Y. Yang X. Wang Y. Bai Y. Xu J. Song H. (2012). Genome sequences of *Brucella melitensis* 16m and its two derivatives 16m1w and 16m13w, which evolved in vivo. *J. Bacteriol.* 194:5489. 10.1128/JB.01293-12 22965104 PMC3457236

[B19] Kirzinger M. W. Stavrinides J. (2012). Host specificity determinants as a genetic continuum. *Trends Microbiol.* 20 88–93. 10.1016/j.tim.2011.11.006 22196375

[B20] Li H. Durbin R. (2009). Fast and accurate short read alignment with burrows-wheeler transform. *Bioinformatics* 25 1754–1760. 10.1093/bioinformatics/btp324 19451168 PMC2705234

[B21] Lucero N. E. Ayala S. M. Escobar G. I. Jacob N. R. (2008). *Brucella* isolated in humans and animals in latin America from 1968 to 2006. *Epidemiol. Infect.* 136 496–503. 10.1017/S0950268807008795 17559694 PMC2870831

[B22] Mailles A. Ogielska M. Kemiche F. Garin-Bastuji B. Brieu N. Burnusus Z. (2017). *Brucella suis* biovar 2 infection in humans in France: emerging infection or better recognition? *Epidemiol. Infect.* 145 2711–2716. 28784192 10.1017/S0950268817001704 PMC9148767

[B23] Office International des Epizooties [OIE] (2016). *Brucellosis (Brucella abortus, B. melitensis and B. suis)(Infection with abortus, B., B. melitensis and B. suis)*. Paris: OIE Terrestrial Manual.

[B24] Page A. J. Cummins C. A. Hunt M. Wong V. K. Reuter S. Holden M. T. (2015). Roary: rapid large-scal prokaryote pan genome analysis. *Bioinformatics* 31 3691–3693. 10.1093/bioinformatics/btv421 26198102 PMC4817141

[B25] Ridler A. L. West D. M. Collett M. G. (2012). Pathology of *Brucella ovis* infection in red deer stags (*Cervus elaphus*). *New Zealand Vet. J.* 60 146–149. 10.1080/00480169.2011.638269 22352933

[B26] Ryall B. Eydallin G. Ferenci T. (2012). Culture history and population heterogeneity as determinants of bacterial adaptation: the adaptomics of a single environmental transition. *Microbiol. Mol. Biol. Rev.* 76 597–625. 10.1128/MMBR.05028-11 22933562 PMC3429624

[B27] Seemann T. (2014). Prokka: rapid prokaryotic genome annotation. *Bioinformatics* 30 2068–2069. 10.1093/bioinformatics/btu153 24642063

[B28] Soler-Llorens P. F. Quance C. R. Lawhon S. D. Stuber T. P. Edwards J. F. Ficht T. A. (2016). A *Brucella* spp. Isolate from a pac-man frog *(Ceratophrys ornata)* reveals characteristics departing from classical *Brucellae*. *Front. Cell Infect. Microbiol.* 6:116. 10.3389/fcimb.2016.00116 27734009 PMC5040101

[B29] Stoffregen C. Olsen S. C. Wheeler C. J. Bricker B. J. Palmer M. V. Jensen A. E. (2007). Diagnostic characterization of a feral swine herd enzootically infected with *Brucella*. *J. Vet. Diagn. Invest.* 19 227–237. 17459850 10.1177/104063870701900301

[B30] Suárez-Esquivel M. Baker K. S. Ruiz-Villalobos N. Hernandez-Mora G. Barquero-Calvo E. Gonzalez-Barrientos R. (2017). *Brucella* genetic variability in wildlife marine mammals populations relates to host preference and ocean distribution. *Genome Biol. Evol.* 9 1901–1912. 10.1093/gbe/evx137 28854602 PMC5554395

[B31] Tamura K. Stecher G. Peterson D. Filipski A. Kumar S. (2013). MEGA6: molecular evolutionary genetics analysis version 6.0. *Mol. Biol. Evol.* 30 2725–2729. 10.1093/molbev/mst197 24132122 PMC3840312

[B32] Tenover F. C. Arbeit R. D. Goering R. V. Mickelsen P. A. Murray B. E. Persing D. H. (1995). Interpreting chromosomal DNA restriction patterns produced by pulsed-field gel electrophoresis: criteria for bacterial strain typing. *J. Clin. Microbiol.* 33 2233–2239. 7494007 10.1128/jcm.33.9.2233-2239.1995 PMC228385

[B33] Thompson J. D. Higgins D. G. Gibson T. J. (1994). CLUSTAL W: improving the sensitivity of progressive multiple sequence alignment through sequence weighting, position-specific gap penalties and weight matrix choice. *Nucleic Acids Res.* 22 4673–4680. 7984417 10.1093/nar/22.22.4673 PMC308517

[B34] Tsolis R. M. Seshadri R. Santos R. L. Sangari F. J. Lobo J. M. de Jong M. F. (2009). Genome degradation in *Brucella ovis* corresponds with narrowing of its host range and tissue tropism. *PLoS One* 4:e5519. 10.1371/journal.pone.0005519 19436743 PMC2677664

[B35] Verger J. M. Garin-Bastuji B. Grayon M. Mahé A. M. (1989). La brucellose bovine à *Brucella melitensis* en France. *Ann. Rech. Vet.* 20 93–102. 2930137

[B36] Wareth G. Melzer F. El-Diasty M. Schmoock G. Elbauomy E. Abdel-Hamid N. (2017). Isolation of *Brucella abortus* from a dog and a cat confirms their biological role in re-emergence and dissemination of bovine brucellosis on dairy farms. *Transbound. Emerg. Dis.* 64 e27–e30. 10.1111/tbed.12535 27307391

[B37] Wattam A. R. Williams K. P. Snyder E. E. Almeida NF Jr Shukla M. Dickerman A. W. (2009). Analysis of ten *Brucella* genomes reveals evidence for horizontal gene transfer despite a preferred intracellular lifestyle. *J. Bacteriol.* 191 3569–3579. 10.1128/JB.01767-08 19346311 PMC2681906

[B38] Wattam A. R. Foster J. T. Mane S. P. Beckstrom-Sternberg S. M. Beckstrom-Sternberg J. M. Dickerman A. W. (2014). Comparative phylogenomics and evolution of the Brucellae reveal a path to virulence. *J. Bacteriol.* 196 920–930. 10.1128/JB.01091-13 24336939 PMC3957692

